# Synergistic Catalytic Effects on Nitrogen Transformation during Biomass Pyrolysis: A Focus on Proline as a Model Compound

**DOI:** 10.3390/molecules29133118

**Published:** 2024-06-30

**Authors:** Shan Cheng, Kehui Yao, Hong Tian, Ting Yang, Lianghui Chen

**Affiliations:** School of Energy and Power Engineering, Changsha University of Science and Technology, Changsha 410114, China; shancheng@csust.edu.cn (S.C.); yaokh1125@163.com (K.Y.); yt1112934@163.com (T.Y.); lianghui20000812@163.com (L.C.)

**Keywords:** biomass pyrolysis, proline, NOx precursors, composite catalysts, synergistic effects

## Abstract

To investigate the control mechanisms of NOx precursors and the synergistic effects of composite catalysts during proline pyrolysis, a systematic series of experiments was conducted utilizing composite catalysts with varying Fe-Ca ratios. Product distribution analysis was employed to elucidate the catalysts’ mechanisms in reducing NOx precursor emissions. The synergistic interactions between Fe and Ca were quantitatively assessed through comparative theoretical and experimental release calculations. The results indicate that an increase in the Fe content in the catalyst led to a rise in amine concentrations from 0.9% to 2.95%, implying that Fe facilitates the generation of amine-N through ring-opening and substitution reactions. When the Fe to Ca ratio was balanced at 1:1, nitrogen predominantly participated in the formation of purines via cyclization and substitution reactions. Additionally, all composite catalysts exhibited a suppressive effect on the release of NOx precursors, attributed to their significant enhancement of solid product retention. Fe-Ca composite catalyst synergistically inhibits the release of gaseous nitrogen. Notably, the strongest synergistic effect was observed with a 1:3 Fe to Ca ratio, which reduced the release of NH_3_ by 38.7% and HCN by 53.6% during proline pyrolysis. This study offers valuable insights into the control of NOx precursors and the optimization of nitrogen-rich biomass pyrolysis processes.

## 1. Introduction

The issues surrounding fossil fuel use, including environmental pollution, climate change, and the energy crisis, are compelling the global community to seek out viable alternatives [1]. Biomass energy stands out with its accessibility, renewability, and eco-friendliness, and as the sole renewable source of carbon-containing compounds, it holds significant potential and value for replacing fossil fuels [2,3]. Pyrolysis of biomass has garnered considerable research attention and practical application. It boasts a high conversion rate, allowing for the simultaneous production of fuels like char, tar, and gas, as well as the targeted generation of high-value platform chemicals [4]. However, a key component of pollution from fossil fuels is nitrogen, and this element also poses a significant challenge for the advancement of biomass energy. The nitrogen content in biomass ranges from approximately 0.3 to 6 wt.%, with protein forms containing as much as 80% nitrogen [5,6]. Proteins are inherently unstable and prone to decompose at elevated temperatures, releasing NH_3_, HCN, etc. These gases will be oxidized into NOx in the subsequent combustion process, leading to serious environmental pollution problems. Consequently, understanding the migration and transformation pathways of nitrogen during pyrolysis is of paramount importance, particularly in managing the formation of NOx precursors [7,8].

Working with raw biomass in research can be quite challenging due to its intricate composition and structure. For instance, carbonyl compounds present in biomass can engage in Maillard reactions with amino compounds, and minerals found in ash can catalyze the secondary decomposition of certain nitrogen-containing intermediates [9]. Thus, to more effectively investigate the pathways of nitrogenous chemical formation, it is crucial to streamline the biomass composition. Amino acids are the fundamental units of proteins, and among them, proline stands out as a typical cyclic amino acid and is the most abundant among nitrogenous heterocyclic amino acids [10]. Proline has been selected by numerous researchers as a model compound for studying nitrogen transformation in biomass. Liu et al. chose proline as the biomass nitrogen model and discovered that tar contained a significant number of amines, and that proline, with its nitrogen-containing ring structure, was prone to forming N-heterocyclic rings [11]. Ren et al. investigated the changes in NOx precursors during the co-pyrolysis of proline and cellulose, noting an increase in NH_3_ release while there was a substantial decrease in HCN release [12]. Therefore, using proline as a nitrogen model compound to study the occurrence of reactions such as condensation, deamination, and aromatization during pyrolysis, as well as the release pathways of nitrogen-containing gases, can significantly enhance our understanding of the nitrogen transformation process in biomass pyrolysis.

Conventional biomass pyrolysis technology is plagued by a few significant issues: it yields a complex mixture of products, has a low conversion rate, and the purification process is quite challenging. These factors severely limit the utility and application scope of the pyrolysis products [13]. However, introducing catalysts into the pyrolysis process can substantially alter the distribution and characteristics of the pyrolysis products. Wheat straw pyrolysis studies have shown that the addition of CaO can inhibit the conversion of nitrogen to NH_3_ and HCN [14]. Ren et al. highlighted that mineral such as potassium carbonate (K_2_CO_3_), calcium oxide (CaO), and iron oxide (Fe_2_O_3_) can shift the distribution of primary pyrolysis products from amino acids like phenylalanine, aspartic acid, and leucine, subsequently impacting the secondary pyrolysis pathway of 2,5-diketopiperazine (DKP) [15]. Tian et al. observed that alkali metals can modify the formation pathway of heterocyclic nitrogen compound in the pyrolysis products of phenylalanine at temperatures as high as 800 °C. Current research on NOx precursors generated by amino acid pyrolysis is predominantly centered on the impact of single catalyst at high temperatures [16]. There is relatively little focus on the effect of catalyst on gaseous nitrogen when the target product of pyrolysis is tar, and even less consideration is given to the synergistic effects that may occur during composite catalysis.

Therefore, this study selected proline as the nitrogen model compound, while Fe_2_O_3_, CaO, and various mixtures of these two oxides served as catalysts. Through comparative analysis of the distribution of nitrogen-containing compounds in gas and tar generated by the pyrolysis of proline at different temperatures and different proportions of Fe_2_O_3_ and CaO, the formation pathway of main nitrogen-containing chemicals was explored. The synergistic effect of Fe and Ca in composite catalysis was emphasized. The results are expected to clarify the regulatory mechanism of Fe_2_O_3_, CaO, and Fe-Ca composite catalysts on nitrogen transformation, which will provide a theoretical basis for controlling NOx emissions during thermal chemical conversion of nitrogen-rich biomass.

## 2. Result and Discussion

### 2.1. Effect of Catalysts at Different Temperatures

#### 2.1.1. Nitrogen Distribution

**Figure 1:**
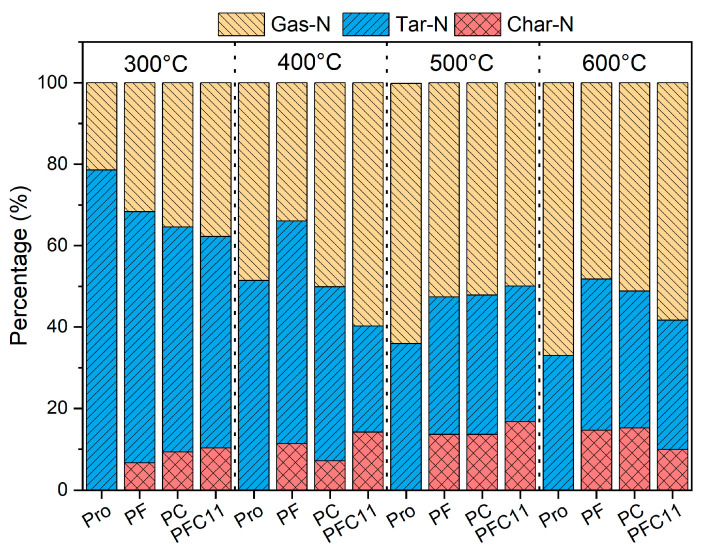
Nitrogen distribution in char, tar, and gas products.

The nitrogen in the pyrolysis products of Pro was converted to the percentage of the total nitrogen before pyrolysis, and the nitrogen balance was shown in Figure 1. Pyrolysis of Pro was conducted over a temperature range of 300 to 600 °C, revealing a significant trend: the yield of tar-N decreased from 78.6% to 33.0%, while the yield of gas-N increased from 21.4% to 77.0%. This trend suggests that Pro readily converts into volatile nitrogen species, with higher temperatures favoring the production of smaller molecular gas-N compounds [17].

After the addition of Fe_2_O_3_, there was a notable shift in the nitrogen distribution. Specifically, the production of char-N increased to a range of 6.7% to 16.6%, while the tar-N yield was correspondingly reduced to between 30.8% and 61.7%. This suggests that Fe may interact with nitrogenous compounds to form char-N, which exhibits high thermal stability [18]. Consequently, this results in an increased nitrogen residue in the char and a subsequent reduction in tar production. Furthermore, at 300 °C, the presence of Fe_2_O_3_ led to a 10.3% increase in gas-N; however, at 400 °C, it resulted in a decrease in gas-N by 11.3–18.8%. This may be related to the fact that Fe can only promote the conversion of NH_3_ into H_2_ and N_2_ at higher temperatures [19,20].

Similar to Fe_2_O_3_, CaO also exhibited an impact on nitrogen conversion. However, CaO was observed to inhibit the production of gas-N exclusively at temperatures above 500 °C. Additionally, the char yield of PC increased from 7.2% to 9.3% at 300 to 400 °C, and further rose to 13.6% to 15.2% at 500~600 °C. These observations suggest that nitrogen was sequestered within the char by Ca, thereby reducing both tar-N and gas-N emissions.

During analysis of Fe-Ca co-catalysis within the 300 to 400 °C range, there was a synergistic effect observed. The yields of char-N and gas-N increased by 1% to 7% and 2.4% to 25.9%, respectively, compared to single catalysis. This indicates that the combined presence of Fe and Ca not only promotes the stable formation of char-N but also catalyzes the decomposition of nitrogenous compounds within the tar. At temperatures exceeding 500 °C, the co-promotion of char-N by Fe and Ca persists. However, at 600 °C, a contrasting trend emerges, with gas-N increasing from 49.9% to 58.4%. This reversal may be due to the gradual formation of calcium ferrite (Ca_2_Fe_2_O_5_) from a small amount of Fe-Ca, which could impede the nitrogen fixation reaction [21].

#### 2.1.2. Release of NH_3_ and HCN

**Figure 2:**
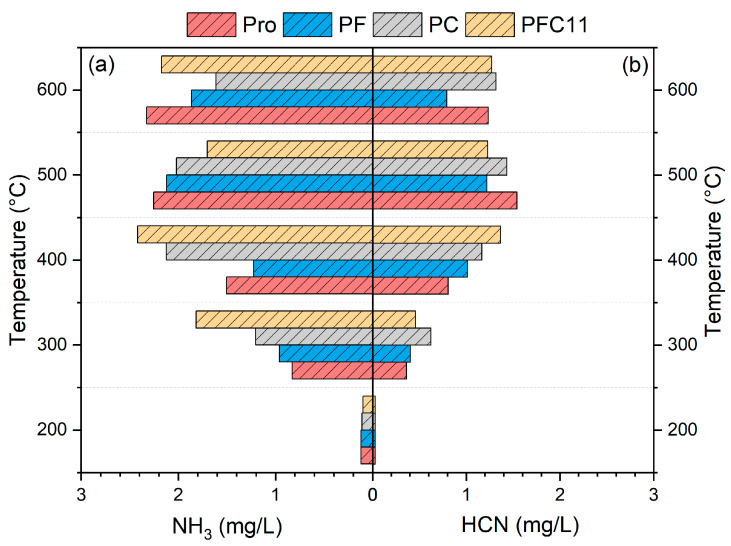
The nitrogen-containing gases (a) NH_3_, (b) HCN release.

Figure 2 illustrates that the release of NH_3_ and HCN increases with increasing temperature. The predominant nitrogen-containing gas produced from Pro pyrolysis is NH_3_, with its release being 1.2 to 4 times that of HCN. The formation of NH_3_ occurs through the cleavage of C-N bonds and the subsequent removal of amino groups, a process that is relatively straightforward compared to the generation of HCN, which necessitates a series of dehydrogenation and dehydration reactions [22].

Upon the addition of Fe_2_O_3_, there is a significant reduction in the release of NH_3_ at temperatures ranging from 400 to 600 °C, by 5.9% to 19.9% relative to Pro. This decrease can be attributed to the catalytic activity of Fe, which facilitates the conversion of NH_3_ into H_2_ and N_2_. In contrast, the reduction in HCN release is less pronounced, decreasing only from 1.22 mg/L to 0.79 mg/L at 500 °C. This minor reduction suggests that the redox reaction between Fe_2_O_3_ and HCN is temperature-dependent [23]. When CaO is introduced, the release of NH_3_ at temperatures above 500 °C is notably reduced by 10.4% to 30.7%. This reduction is primarily due to the altered catalytic effect of CaO, which promotes the dimerization of amine-N molecules and their subsequent incorporation into tar [24]. The pattern of HCN release from PC is consistent with that observed with Fe_2_O_3_, albeit with slightly higher release amounts. This can be explained by the higher oxidizing nature of Fe_2_O_3_ compared to CaO, making it more reactive with HCN. Compared with Pro, the change of Fe-Ca is more similar to CaO. The changes are more pronounced at 500 °C, where the release of NH_3_ is reduced by 24.4% and that of HCN by 20.2%. The effect of Ca on Fe-Ca composite is greater.

#### 2.1.3. Formation of N-Containing Compounds

**Figure 3:**
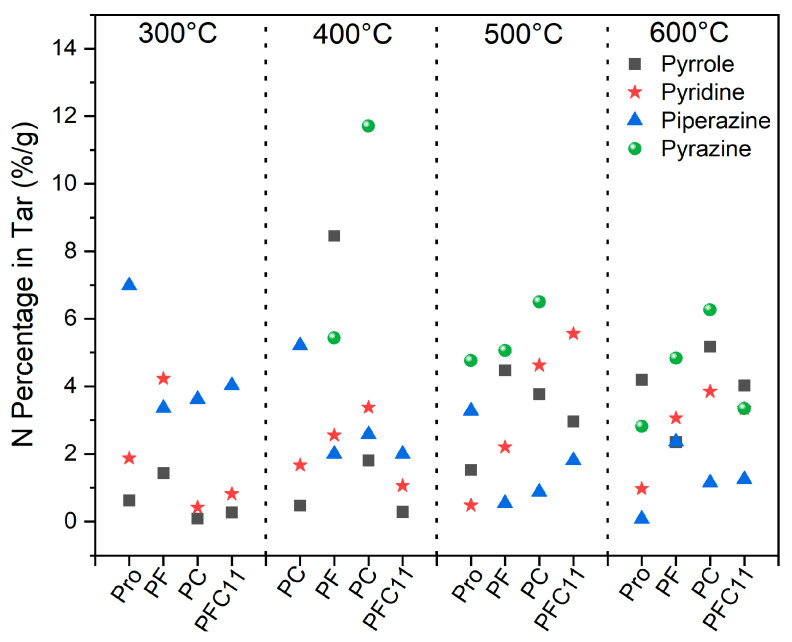
Effect of Fe and Ca on the production of pyrrole, pyridine, piperazine, and pyrazine.

**Figure 4:**
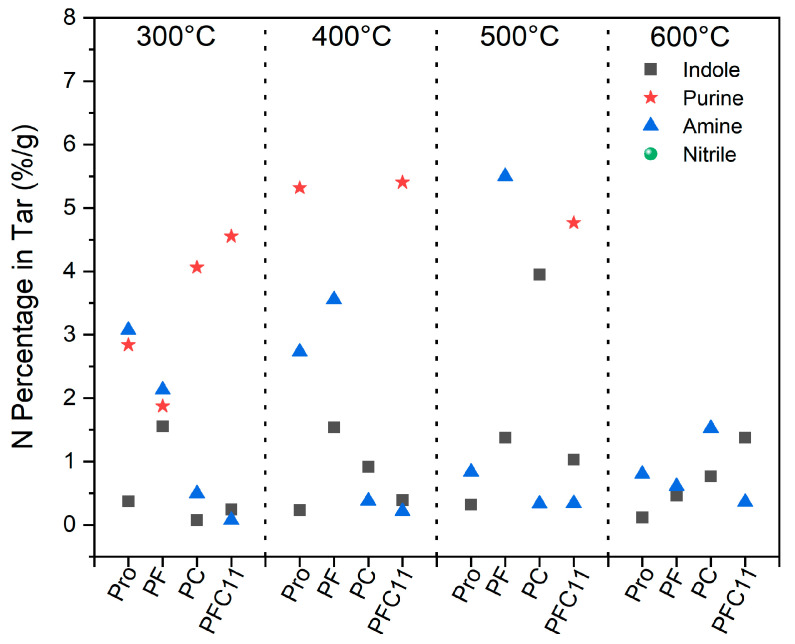
Effect of Fe and Ca on the production of indole, purine, amine, and nitrile.

Figure 3 and Figure 4 depict the peaks of all volatile products identified as pyrrole, pyridine, piperazine, pyrazine, indole, purine, amines, and nitriles based on their peak areas [25]. Initially, at 300 °C, the tar-N of Pro was predominantly composed of piperazine, amines, and purines, accounting for 6.89%, 3.07%, and 2.84%, respectively. As the temperature rose to 400 °C, there was a noticeable shift in the composition: the contents of amines and piperazine decreased to 2.73% and 5.22%, respectively, while the yield of purines increased to 5.32%. Upon further temperature increase to 500 °C, purine compounds were virtually absent, with pyrazine and pyrrole emerging as the predominant species. This suggests that piperazine and purine likely serve as intermediate products in the pyrolysis of Pro [11]. In the later stage of pyrolysis, between 500 °C and 600 °C, pyrazine (4.75%) and pyrrole (4.2%) were identified as the main products. Pyrazine is hypothesized to originate from the dehydrogenation of piperazine, while pyrrole may form through a series of reactions involving the decarboxylation and dehydrogenation of Pro, or through a dehydration step followed by a secondary decarboxylation [26,27].

The distribution of main tar-N is illustrated in Figure 3. Upon the addition of Fe_2_O_3_, the content of piperazine was observed to decrease to a range of 0.54% to 3.61% at temperatures between 300 °C and 500 °C, with pyrazine emerging as a notable product at 400 °C. This observation suggests that Fe can facilitate the dehydrogenation of piperazine, thereby converting it into pyrazine. At 300 °C, the content of pyridine peaked at a maximum of 4.2%, and at 400 °C, pyrrole reached its maximum content of 8.5%. Pyridine is believed to form from Pro through a series of reactions including ring-opening, substitution, dehydrogenation, and Diels–Alder reactions [28]. It is proposed that Fe enhances these reactions, with its promotional effect on pyridine formation being particularly pronounced at the lower temperature, such as 300 °C. After the addition of CaO, the behavior of pyrazine and piperazine was found to be consistent with that observed with Fe_2_O_3_. However, the key difference was noted in the content of pyrrole and pyridine, which peaked at 5.17% at 600 °C and 4.6% at 500 °C, respectively. This suggests that at higher temperatures with CaO present, amine groups undergo condensation reactions, ultimately leading to the formation of aromatic nitrogen-containing compounds [29].

When Fe_2_O_3_ and CaO were co-added, the yield of piperazine was found to closely resemble that observed with CaO alone, particularly at 300 °C. This suggests that Ca has a competitive advantage over Fe in the conversion of Pro to piperazine at lower temperatures. However, the formation of pyrazine was not detected within the temperature range of 400 °C to 500 °C. This absence indicates that the presence of both Fe and Ca may inhibit the conversion of piperazine to pyrazine through a series of reactions such as ring-opening, deoxidation, and dehydrogenation. This inhibitory effect could be attributed to an antagonistic interaction between Fe and Ca. Upon increasing the temperature to 600 °C, the antagonistic effect between Fe and Ca appears to diminish, leading to a pyrazine yield of 3.3%, which is comparable to that of Pro alone. During the pyrolysis of the Fe-Ca mixture (PFC11), the content of pyrrole and pyridine was found to be similar to that during the pyrolysis of Pro with CaO (PC) alone. However, the promotional effect of Fe-Ca on pyrrole formation was observed to be reduced.

Therefore, the addition of Fe_2_O_3_ and CaO promotes the production of monocyclic compounds such as pyrrole, pyridine, and pyrazine. At 300 °C, Fe primarily enhances the formation of pyridine, while between 400 °C and 600 °C, Ca predominantly promotes the formation of pyrazine. When both Fe_2_O_3_ and CaO are added, there is a significant decrease in pyrrole, pyridine, and piperazine at 300 °C to 400 °C, followed by an increase in pyrrole and pyridine at 500 °C to 600 °C, with the promotional effect on pyrrole formation being reduced.

Both indole and purine are characterized by their polycyclic structures, and their formation and transformation during the pyrolysis of Pro are influenced by the presence of catalysts such as Fe_2_O_3_ and CaO. As shown in Figure 4, these catalysts have contrasting effects on purine yield at 300 °C. The addition of Fe_2_O_3_ significantly reduced the yield of purine to 1.88%. In contrast, CaO increased the yield of purine substantially to 4.06%. However, purine was undetected in the pyrolysis products at temperatures ranging from 400 °C to 600 °C, suggesting that purine acts as an intermediate in the pyrolysis process of Pro. In terms of indole production, both Fe and Ca enhance its formation, with Fe_2_O_3_ increasing indole content to a range of 0.46% to 1.56%, and CaO further elevating indole production to a range of 0.77% to 3.95%. Amine compounds were predominantly found at lower temperatures, indicating that they are likely intermediates in the pyrolysis of Pro. The absence of nitrile in the products suggests that the formation of cyanogen compounds is not favored under these pyrolysis conditions. Specifically, at 300 °C, Fe_2_O_3_ had a negligible effect on amine production. However, between 400 °C and 500 °C, the yield of amines rose to between 3.56% and 5.5%, indicating a promotional effect by Fe_2_O_3_ on amine generation [30]. At 600 °C, CaO exhibited an opposing effect, potentially inhibiting amine production or promoting their further conversion.

When Fe_2_O_3_ and CaO were co-added, purine emerged as the principal product at 300 °C, with a content of 4.55%, which is in line with the yield observed when CaO is used alone. This suggests that at lower temperatures, CaO outperforms Fe_2_O_3_ in promoting the formation of purines, likely through facilitating intermolecular dehydration and dehydrogenation reactions. Prior analyses have established purine as an intermediate product in the pyrolysis of Pro, with its presence diminishing after 400 °C in the rapid pyrolysis products of both Fe_2_O_3_ (PF) and CaO (PC) treated samples. However, in the case of the Fe_2_O_3_ and CaO co-addition (PFC11), purine remains the main product at 400~500 °C, with a yield peak of 5.4% at 400 °C. This indicates that the temperature window for purine formation, which is promoted by CaO, can be broadened by the concurrent presence of Fe_2_O_3_. Furthermore, when both Fe and Ca are added, there is an increase in indole yield by 0.16% to 1.25%, demonstrating a synergistic promotional effect on indole formation. The amine content in the PFC11 samples, ranging from 0.07% to 0.36%, is lower than that in the original samples (0.8% to 3.07%) and the composite samples (0.33% to 5.5%). This suggests that Fe_2_O_3_ can work in conjunction with CaO to enhance the decomposition and transformation of amine [24].

### 2.2. Effects of Different Ratios of Composite Catalysts

#### 2.2.1. Nitrogen Distribution

**Figure 5:**
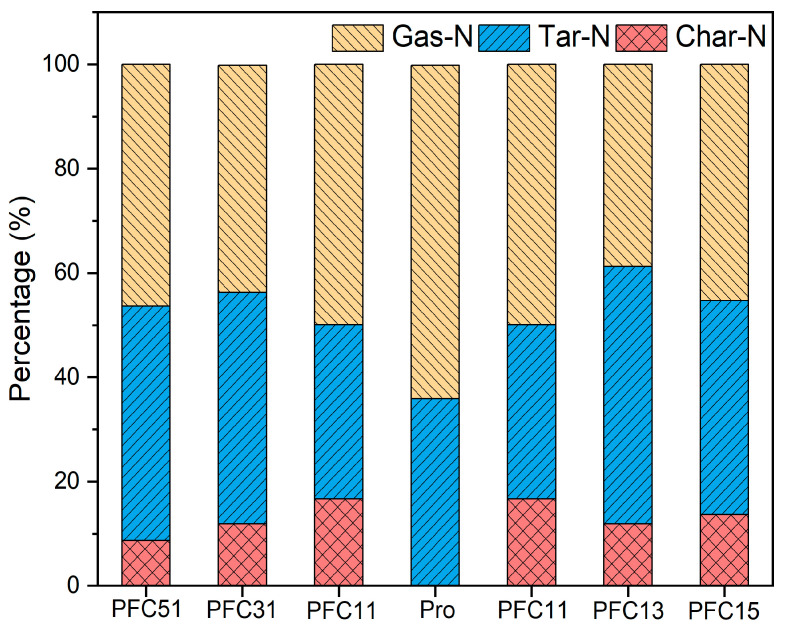
Nitrogen distribution during composite catalysis pyrolysis.

The results previously discussed indicate that the suppression of NH_3_ and HCN formation was most significant at 500 °C when the Fe-Ca catalyst was at a 1:1 ratio. Therefore, the impact of the Fe to Ca ratio in the composite catalysts on the distribution of nitrogen-containing products at 500 °C was further investigated, with the nitrogen balance shown in Figure 5. Notably, the Pro sample did not produce char-N, whereas other samples contained char-N ranging from 8.7% to 16.7%. This suggests that the incorporation of Fe and Ca composite catalysts promotes the formation of pyrolysis char-N, with the 1:1 ratio demonstrating the most substantial effect. As the proportion of Fe and Ca in the catalyst increased, the tar-N content also showed a slight rise, mirroring the observation that tar-N levels in PF and PC are higher than those in PFC11, as illustrated in Figure 1. The Fe and Ca composite catalysts exhibited a high degree of uniformity, leading to a reduction in gas-N formation by 13.95% to 22.97%. When the Fe to Ca ratio was 1:1, the fixation of nitrogen in char was most effective, accounting for 16.7%, thereby reducing tar-N and gas-N. However, at a Fe-Ca ratio of 1:3, the lowest gas-N content was recorded at 40.9%, while the highest tar-N content reached 49.4%. This suggests that this ratio is more conducive to retaining nitrogen in the tar, resulting in a decrease in gas-N. In summary, the Fe-Ca composite catalysts effectively inhibited the precipitation of gas-phase products, with the most significant inhibitory effect observed at a Fe-Ca ratio of 1:3.

#### 2.2.2. Release of NH_3_ and HCN

**Figure 6:**
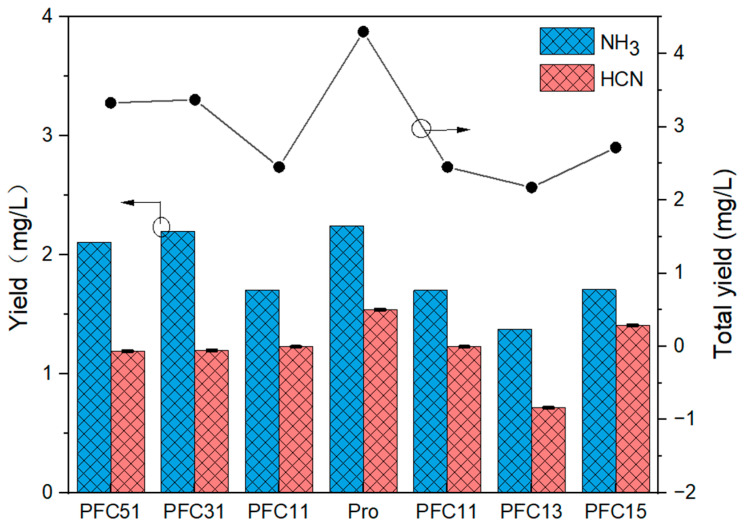
The nitrogen-containing gases release during composite catalysis pyrolysis.

As shown in Figure 6, the release of NH_3_ is 1.2 to 1.9 times higher than that of HCN. The addition of composite catalysts has effectively reduced the release of NH_3_ from 2.24 mg/L to a range of 1.3 to 2.1 mg/L, and HCN from 1.54 mg/L to a range of 0.67 to 1.41mg/L. At an Fe to Ca ratio of 1:1, the formation of NH_3_ and HCN was inhibited by 24.4% and 20.3%, respectively. When the Fe to Ca ratio exceeded 1, indicating Fe dominance, the PFC51 and PFC31 catalysts exhibited minimal deviation from the Pro sample, with a mere reduction in NH_3_ and HCN emissions by 0.04~0.1 mg/L and 0.3~0.4 mg/L, respectively. Conversely, when the ratio was less than 1, favoring Ca, PFC13 and PFC15 catalysts more closely resembled PFC11, achieving a reduction in NH_3_ and HCN emissions by 0.5~0.9 mg/L and 0.1~0.8 mg/L, respectively. The dominance of Fe had a negligible impact on NH_3_ emissions, aligning with the observation in Figure 2 that Fe only marginally reduces NH_3_ levels. In contrast, a Ca-rich environment was more effective in curbing NH_3_ emissions, with the most pronounced synergistic control effect observed at a 1:3 ratio.

Considering that NH_3_ mainly generated from deamination reaction, while HCN arises predominantly from the decomposition of nitrile-N or the ring-opening of heterocyclic-N [31]. Both Fe and Ca are capable of interacting with volatile nitrogen compounds, potentially sequestering nitrogen in char via the formation of MeCxNy and facilitating the cyclization or dehydrogenation of amine in tar to yield nitrile. Therefore, the most potent synergistic effect was observed at a 1:3 Fe to Ca ratio, which could be attributed to two factors. Firstly, this ratio fostered the mutual conversion of nitrogenous compounds within tar. Secondly, CaO was particularly effective at inhibiting NH_3_ release, and the concomitant addition of Fe_2_O_3_ could react with a minor amount of CaO to form Ca_2_Fe_2_O_5_, and high alkalinity was more conducive to the occurrence of sintering [21,32]. Therefore, the optimal Fe-Ca ratio for Gas-N control was 1:3.

#### 2.2.3. Formation of N-Containing Compounds

**Figure 7:**
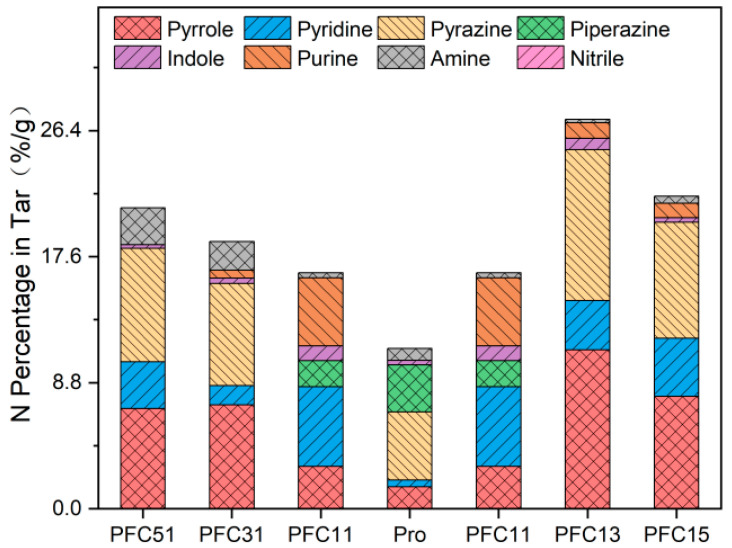
Distribution of nitrogen products in tar during composite catalysis pyrolysis.

The classification of nitrogen species in tar follows the previously described method. As depicted in Figure 7, the pyrolysis of Pro resulted in a tar-N content of 11.67%. In contrast, the tar-N contents for PFC51, PFC31, PFC11, PFC13, and PFC15 were 21.0%, 18.6%, 16.5%, 27.2%, and 21.8%, respectively. In the pyrolysis tar-N, piperazine (3.42%), pyrazine (4.96%), and pyrrole (1.59%) are identified as the predominant nitrogenous compounds. Compared to Pro, PFC11 not only facilitated the production of pyrrole and pyridine but also significantly promoted the formation of polycyclic compounds, such as purine (increased from 0% to 4.8%) and indole (from 0.3% to 1%). Upon altering the Fe to Ca ratio in the composite catalysts, there was a notable increase in pyridine, pyrrole, and pyrazine compared to Pro. However, when compared to PFC11, purine, piperazine, and pyridine experienced a decrease ranging from 3.7~4.8%, 1.8%, and 1.5~4.2%, respectively. This suggests that when the Fe to Ca ratio deviates from 1, the impact of the composite catalysts more closely resembles that of a single catalyst, favoring the production of mono-ring products. A high proportion of Ca in the catalysts, specifically CaO, can promote the dimerization of amine-N molecules, thereby enhancing the inhibition of NH_3_ emissions and strengthening the synergistic effect of the Fe-Ca composite catalysts [24]. Among the composite catalysts, PFC13 demonstrated the most pronounced synergistic effect, significantly enhancing the production of pyrazine (10.6%), pyridine (3.4%), and pyrrole (11.1%). This is attributed to the Fe-Ca composite catalysis promoting the dehydrogenation of Pro and other reactions that lead to the formation of monocyclic substances [33,34].

### 2.3. Synergistic Effect of Composite Catalysts

**Figure 8:**
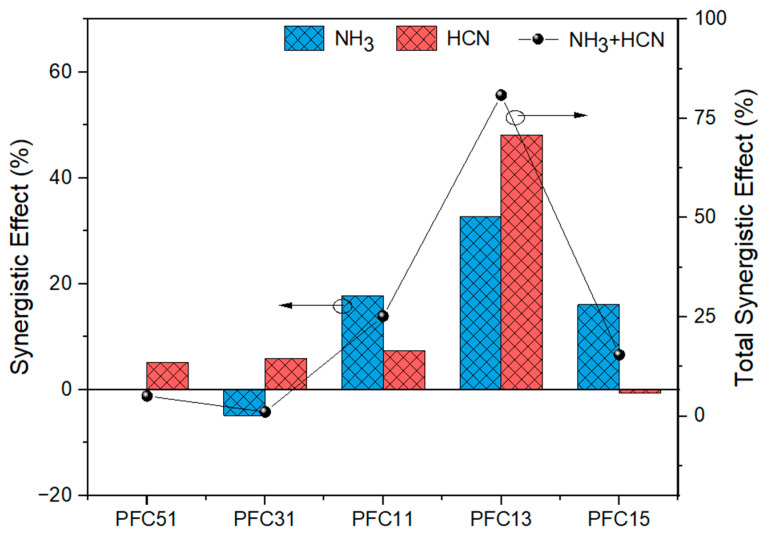
Synergistic effect of composite catalysts.

The synergistic effect is quantified as the percentage difference between the theoretical release and the actual release of nitrogen compounds during the catalytic pyrolysis process. The theoretical release is based on the individual releases from single catalysis, and a positive synergistic effect indicates that the Fe-Ca composite catalyst synergistically inhibits the release of gaseous nitrogen. As depicted in Figure 8, the Fe-Ca composite catalysts exhibit a synergistic inhibitory effect on both NH_3_ and HCN emissions. Notably, ratios other than 1:3 show a more pronounced inhibitory effect on NH_3_, while the 1:3 ratio has a more significant impact on HCN. At a 3:1 ratio, an antagonistic effect is observed, promoting the release of NH_3_. The synergistic effect increases and then diminishes with an increasing proportion of Ca, reaching its peak at a 1:3 ratio, where it enhances the actual control effect by 80.9% over the theoretical expectation.

The results shown in Figure 7 indicate Fe can react with amino-N, which is more susceptible to decomposition and subsequent NH_3_ production than other nitrogenous macromolecules. Consequently, as the proportion of Ca in the composite catalysts increases, a more pronounced synergistic effect is observed on the release of NH_3_. When the Fe-Ca ratio is further increase to 1:5, it is hypothesized that a high-alkali environment may facilitate the interring of Fe-Ca to form Ca_2_Fe_2_O_5_ [32]. This could lead to a loss of reactivity with nitrogenous compounds, thereby reducing the nitrogen fixation effect.

### 2.4. Effect of Composite Catalysts on Pyrolysis Paths

**Figure 9:**
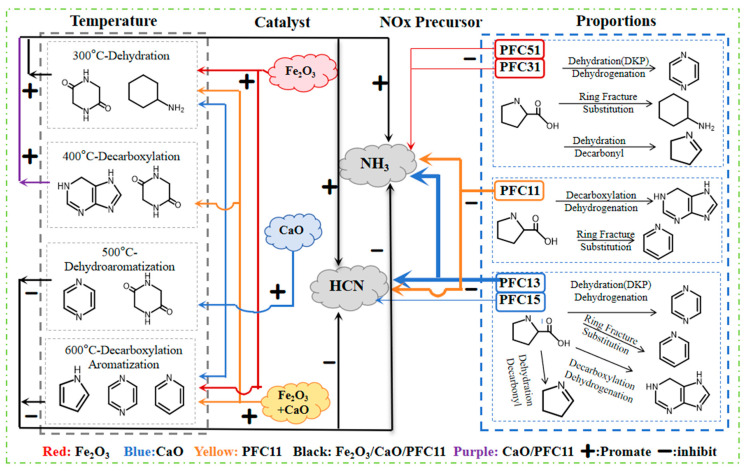
Influence paths of Fe and Ca on the formation of nitrogen-containing products (arrow represents the direction of influence, line thickness represents intensity).

Amino acid pyrolysis initiates through four primary reactions: decarboxylation, dehydrogenation, dehydration, and deamination [35,36]. Specifically for Pro, the predominant reaction is decarboxylation, leading to the formation of pyrrolidine. Subsequent dehydrogenation of pyrrolidine yields pyrrole. As illustrated on the left side of Figure 9, DKP is generated through a double dehydration reaction between Pro molecules [37], representing one of the primary products during the initial phase of pyrolysis at 300 °C. Upon increasing the temperature to 400 °C, purine emerges as the principal product, constituting 5.32% of the pyrolysis products. In the later stages of pyrolysis (500~600 °C), pyrazine (4.76%) and pyrrole (4.19%) become the main products. Pyrazine originates from the ring-opening and dehydrogenation of piperazine, while pyrrole is produced directly from Pro through a sequence of decarboxylation and dehydrogenation [26]. Finally, Pro also undergoes deamination, where the carbon-nitrogen single bond within the heterocyclic amine breaks down under heat. This bond rupture facilitates the combination with hydrogen radicals and other species, culminating in the formation of NH_3_ [38].

The addition of Fe_2_O_3_, CaO, and their combination (Fe_2_O_3_+CaO) has been shown to enhance the cyclization and dehydrogenation processes of Pro, thereby facilitating the synthesis of monocyclic heterocyclic nitrogen-containing compounds. When Fe_2_O_3_ and CaO were used individually, they notably increased the yield of monocyclic compounds such as pyrazine, pyrrole and pyridine. Specifically, at 300 °C, the presence of Fe_2_O_3_ was found to favor the production of piperazine, whereas at temperatures ranging from 400 to 600 °C, CaO was more effective in promoting the formation of piperazine. Fe_2_O_3_ also showed a beneficial effect on the formation of indole and pyridine across the temperature range of 300 to 600 °C. At 300 °C, the intermediate pyrrolidine can be converted into purine, while at higher temperatures (400~600 °C), the conditions are more favorable for the generation of pyrazine and pyridine.

When Fe_2_O_3_ and CaO were combined, CaO played a leading role in the early stage and significantly promoting the formation of purine. Furthermore, the presence of Fe_2_O_3_ extended the temperature window in which purine formation was enhanced by CaO. At the higher end of the temperature range, specifically between 500 and 600 °C, the combined effect of Fe_2_O_3_ and CaO synergistically promoted the formation of pyridine, which indicating that they worked together to enhance the decarboxylated dehydrogenation of Pro following ring-opening. Additionally, the release of gaseous nitrogen, as depicted in Figure 2, reveals that the Fe-Ca combination had a pronounced promotional effect on the release of NH_3_ and HCN at temperatures between 300~400 °C. However, as the temperature increased, this promotional effect diminished, eventually leading to a noticeable inhibitory effect on the release of these gases.

The right side of Figure 9 illustrates the impact of composite catalyst ratios on the transformation of nitrogen-containing products during the pyrolysis of Pro. The combined addition of Fe_2_O_3_ and CaO facilitates key reactions such as decarboxylation, substitution, and ring-opening of Pro, which in turn enhances the production of polycyclic compounds like indole and purine. When Fe predominates in the composite catalyst, the types of main products resemble those obtained from non-catalytic pyrolysis of Pro, with monocyclic nitrogen-containing compounds such as pyrrole and pyrazine remaining the primary constituents. When compositing with Ca, the yield of amine-N escalates as the proportion of Fe in the composite catalyst increases. CaO, on the other hand, reacts with amine-N, effectively fixing nitrogen in char. As the proportion of Fe in the composite catalysts rises, its promotional effect on amine-N formation surpasses the nitrogen-fixing effect of Ca. Since amines can deaminate to produce NH_3_, an increase in the proportion of Fe leads to a reduced inhibitory effect on NH_3_ production. At a 1:1 ratio of Fe to Ca in the composite catalysts, the catalysts primarily exhibit the catalytic influence of Ca, resulting in products predominantly formed from purine and pyridine, with Fe’s promotional effect on pyrrole also being evident. If the proportion of Ca is further increased, its leading role becomes more pronounced, which significantly enhances the depolymerization, ring-opening, and dehydrogenation reactions of Pro [39]. This leads to an increased formation of pyrazine, pyridine, and purine. The most pronounced synergistic inhibitory effect on the release of NH_3_ and HCN is observed when the ratio of Fe to Ca is adjusted to 1:3. This suggests an optimal balance where the combined action of Fe and Ca maximizes the desired transformations while minimizing the formation of undesired nitrogenous byproducts.

## 3. Materials and Methods

### 3.1. Sample Preparation

The chemicals were purchased from Sinopharm Chemical Reagent Co., Ltd. (Shanghai, China). The mass ratio of amino acid and catalyst was 1:1, and the mass ratios of Fe_2_O_3_ and CaO in the composite catalysts were 5:1, 3:1, 1:1, 1:3, and 1:5. After the amino acid and catalyst were mixed, they were ground in an agate mortar, and then dried for the experiment [40]. Proline was labeled as Pro, while proline added to Fe_2_O_3_ or CaO were denoted as PF and PC, respectively. Proline added to composite catalysts were labeled as PFC51, PFC31, PFC11, PFC13, and PFC15, according to the mass ratios of Fe_2_O_3_ and CaO of 5:1, 3:1, 1:1, 1:3 and 1:5, respectively.

### 3.2. Pyrolysis Experiments

Pyrolysis experiments were performed in a horizontal tube furnace system. High purity argon (purity > 99.999%) was used as carrier gas with a flow rate of 40 mL/min. A sample of 1 g was taken for pyrolysis experiment. The pyrolysis temperature range was 300~600 °C, the heating rate was 15 °C/min, and the temperature was maintained at 500 °C for 30 min. After each experiment, the char was cooled and collected in an argon atmosphere. The tar was collected by a U-shaped tube immersed in a mixture of ice water, where NH_3_ and HCN were absorbed by 0.1 mol/L H_2_SO_4_ and 0.2 mol/L NaOH solutions, respectively [41]. The experiment was repeated 2~3 times for each sample. After every experiment, the apparatus was heated to 900 °C to burn out the possible residues of tar and soot for subsequent use.

### 3.3. Analysis Methods

The tar was analyzed by GC-MS (Agilent 7890B-5977A, Santa Clara, CA, USA), and the test conditions were consistent with those previously used by our research group [5]. In the MS part, EI ion source was used, sample products were collected by Scan method, and chromatographic peaks were identified and analyzed by NIST mass spectrometry library. The standard solution and detection solution of ammonium and cyanate ions were configured according to the national standards “HJ536-2009” and “HJ484-2009”, respectively [42,43]. The NH4^+^ and CN^−^ absorbed by the solution were measured by the corresponding wave number using a spectrophotometer and converted into the corresponding ion concentration by calculation. The tar yield was taken into account when calculating the distribution of nitrogenous products, which was further converted by the same proline content in the sample. The mass concentration of NH_3_ and HCN in solution was calculated by Equations (1) and (2), respectively:

(1) $$\rho_{N1}=\frac{A_{s}-A_{0}-a}{b\times V_{1}}\times D$$

(2) $$\rho_{N2}=\frac{A_{s}-A_{0}-a}{b}\times \frac{V_{1}}{V_{2}\times V}$$

where $\rho_{N1}$ represents the mass concentration of NH^4+^ in solution, *A* represents the sample selected for the experiment., *A_s_* represents the absorbance of the sample, *A*_0_ represents the absorbance of blank experiment, a and b represent calibrate the intercept and slope of the curve, respectively, *V* represents raw sample volume before distillation, *D* represents the dilution of the sample, $\rho_{N2}$ represents the mass concentration of CN^−^, *V*_1_ represents the selected sample “*A*” volume, and *V*_2_ represents the sample “*A*” volume of the color comparison.

### 3.4. Synergistic Effect Evaluation Methods

For composite catalysis, the theoretical release of NH_3_ and HCN during pyrolysis was calculated by Equation (3), and the synergistic value of the composite catalysts was calculated by Equation (4) [44,45].

(3) $$Y_{Theo}=\frac{x_{1}Y_{1}+x_{2}Y_{2}}{x_{1}+x_{2}}$$

(4) $$Synergistic\;Effect=\frac{Y_{Theo}-Y_{Exp}}{Y_{Theo}}\times 100\%$$

where *Y_Theo_* represents the theoretical release amount of NH_3_ (or HCN) during composite catalysis, *Y*_1_ and *Y*_2_ are the release amount of NH_3_ (or HCN) when Fe_2_O_3_ and CaO are used alone, respectively, *x*_1_ and *x*_2_ are the percentage of Fe_2_O_3_ and CaO in the composite catalysts, respectively, and *Y_Exp_* is the actual release of NH_3_ (or HCN) during the composite catalysis.

## 4. Conclusions

This study systematically compared the control mechanism of NOx precursors produced by the pyrolysis of Pro and the synergistic effect of composite catalysts. The conclusions are as follows:

For composite catalysis, CaO was more competitive in the early stage, so the intermediate product purine increased to 5.4% at 300~500 °C. When Fe_2_O_3_ was present, it extended the temperature range where CaO promotes the formation of purine. At 500~600 °C, the synergistic promotion effect of the two on pyridine was demonstrated, and the pyridine production was increased to 3.4~11.7 times of the original Pro.

When the ratio of Fe to Ca is 1:3, the formation of gas-N is inhibited by 23.0% by retaining nitrogen in tar. With the increase of Fe_2_O_3_ content in the composite catalyst, Fe and Ca showed an antagonistic effect on the formation of NH_3_, because Fe was conducive to the formation of amine-N by reaction such as ring-opening substitution of Pro, and then decomposition to produce NH_3_, reducing the fixation effect of Ca on amine-N.

As the ratio of Ca in the Fe-Ca composite catalysts increased, the synergistic inhibitory effect on NH_3_ first rises and then falls. When the iron-to-calcium ratio was 1:1 and 1:3, Fe and Ca both exhibited a synergistic inhibitory effect on NH_3_ and HCN. At a ratio of 1:3, the strongest synergistic effect was observed, suppressing 38.7% of NH_3_ and 53.6% of HCN.

It should be noted that although this study focused on the synergistic catalytic effects on proline, it is necessary to explore the influence of the other components of the biomass on the nitrogen transformation. In addition, the effect of ash or other inorganic components in biomass on the catalytic effect of Fe_2_O_3_ and CaO is also worth further consideration. Finally, more studies on the pyrolysis of amino acids with different molecular structures should be carried out to reach the conclusion of the universality of nitrogen conversion in biomass pyrolysis, and the targeted regulation strategies of nitrogen-containing substances in biomass should be proposed accordingly.

## Data Availability

Data are available upon request.
